# Effects of Firing Variability on Network Structures with Spike-Timing-Dependent Plasticity

**DOI:** 10.3389/fncom.2018.00001

**Published:** 2018-01-23

**Authors:** Bin Min, Douglas Zhou, David Cai

**Affiliations:** ^1^Center for Neural Science, Courant Institute of Mathematical Sciences, New York University, New York, NY, United States; ^2^NYUAD Institute, New York University Abu Dhabi, Abu Dhabi, United Arab Emirates; ^3^School of Mathematical Sciences, MOE-LSC, Institute of Natural Sciences, Shanghai Jiao Tong University, Shanghai, China

**Keywords:** STDP, linear response theory, correlation structure, firing variability, synaptic plasticity

## Abstract

Synaptic plasticity is believed to be the biological substrate underlying learning and memory. One of the most widespread forms of synaptic plasticity, spike-timing-dependent plasticity (STDP), uses the spike timing information of presynaptic and postsynaptic neurons to induce synaptic potentiation or depression. An open question is how STDP organizes the connectivity patterns in neuronal circuits. Previous studies have placed much emphasis on the role of firing rate in shaping connectivity patterns. Here, we go beyond the firing rate description to develop a self-consistent linear response theory that incorporates the information of both firing rate and firing variability. By decomposing the pairwise spike correlation into one component associated with local direct connections and the other associated with indirect connections, we identify two distinct regimes regarding the network structures learned through STDP. In one regime, the contribution of the direct-connection correlations dominates over that of the indirect-connection correlations in the learning dynamics; this gives rise to a network structure consistent with the firing rate description. In the other regime, the contribution of the indirect-connection correlations dominates in the learning dynamics, leading to a network structure different from the firing rate description. We demonstrate that the heterogeneity of firing variability across neuronal populations induces a temporally asymmetric structure of indirect-connection correlations. This temporally asymmetric structure underlies the emergence of the second regime. Our study provides a new perspective that emphasizes the role of high-order statistics of spiking activity in the spike-correlation-sensitive learning dynamics.

## Introduction

Spike-timing-dependent plasticity (STDP) (Markram et al., 1997; Bi and Poo, 1998; Caporale and Dan, 2008) is one of the major forms of Hebbian learning. In canonical STDP, a synapse is potentiated (depressed) when the presynaptic spikes precede (follow) the postsynaptic ones. With this temporally asymmetric learning window, STDP can regulate both the mean rate and variability of postsynaptic firing (Song et al., 2000), enforce the competition between convergent synaptic inputs (Song et al., 2000), and support temporal sequence learning (Rao and Sejnowski, 2001).

While STDP of a single neuron with many synapses has been well characterized, issues related to the STDP learning dynamics in recurrent networks are yet to be fully addressed, in spite of recent progress (Morrison et al., 2007; Clopath et al., 2010; Gilson, 2010; Kozloski and Cecchi, 2010; Babadi and Abbott, 2013, 2016; Litwin-Kumar and Doiron, 2014; Ocker et al., 2015; Zenke et al., 2015; Bi and Zhou, 2016; Ravid Tannenbaum and Burak, 2016). The role of the low-order statistical structure (i.e., firing rate) of spiking activity has been intensively investigated in the learning dynamics in recurrent networks (Morrison et al., 2007; Kozloski and Cecchi, 2010; Babadi and Abbott, 2013). Though it is known that, as a correlation-sensitive learning rule, STDP inherently incorporates the high-order statistics of spiking activity (Morrison et al., 2008), the role of the high-order statistics of spiking activity in STDP learning dynamics for recurrent networks remains largely unknown. In this work, we explore the issue of how the high-order firing statistics (for example, firing variability) affect the network connectivity structures learned through STDP in recurrent networks. We demonstrate that the information of firing variability is encoded in a linear response kernel that quantifies how a neuron responds to a weak incoming signal. By developing a self-consistent linear response theory pioneered in Pernice et al. (2012), Trousdale et al. (2012), and Helias et al. (2013), we show that this response kernel, together with the firing rate, determines the pairwise correlation of spiking activity. By decomposing the pairwise correlation into one component associated with local direct connections and the other associated with indirect connections, we can identify two distinct dynamical regimes. In the first regime, the local direct connections dominate over the indirect connections in determining the learned network structure. This regime corresponds to those studied in Babadi and Abbott (2013), Kozloski and Cecchi (2010), and Morrison et al. (2007), where the connections from neurons of higher firing rate to those of lower firing rate are strengthened whereas the connections with the opposite situation are weakened. The other is a regime in which the indirect connections dominate over the direct connections in learning dynamics. In this second regime, the connections from neurons of higher firing rate and higher firing variability to those of lower firing rate and lower firing variability are weakened whereas the connections with the opposite situation (i.e., connections from neurons of lower firing rate and lower firing variability to those of higher firing rate and higher firing variability) are strengthened. We find that there is a temporally asymmetric structure of common-input-induced spike correlations between different neuronal populations. This structure is induced by the heterogeneity of firing variability across neuronal populations, which underlies this counterintuitive dynamical regime. Our study provides a theoretical framework for addressing the question of how the high-order statistics of spiking activity affect network structures learned through STDP. Using this framework, we demonstrate how the introduction of heterogeneity of firing variability across different neuronal populations can give rise to counterintuitive network connectivity structures in a plastic neuronal network.

## Methods

### Model

The model we used here is the current-based leaky integrate-and-fire (LIF) model with finite synaptic time constant. It is the same as that in Babadi and Abbott (2013).

(1) $$\begin{array}{r} \tau_{\text{m}}\frac{dV_{i}}{dt}=\left(V_{\text{r}}-V_{i}\right)+I_{i}, \end{array}$$

(2) $$\begin{array}{r} \frac{dI_{i}}{dt}=-\frac{I_{i}}{\tau_{\text{s}}}+\overset{N_{\text{E}},N_{\text{I}}}{\underset{j=1}{\sum }}w_{ij}s_{j}\left(t\right)+\frac{\mu_{\text{ext},i}}{\tau_{\text{s}}}+\frac{\sigma_{\text{ext},i}}{\tau_{\text{s}}}\sqrt{\tau_{\text{m}}}\xi_{i}\left(t\right), \end{array}$$

where τ_m_ = 20 ms is the membrane time constant, *V*_r_ = −60 mV is the resting potential, τ_s_ = 5 ms is the synaptic time constant, *w*_*ij*_ is the synaptic strength from neuron *j* to neuron *i* and *s*_*j*_(*t*) is the spike train of neuron *j*. The parameters μ_ext,*i*_ and $\sigma_{\text{ext},i}^{2}$ are the mean and variance of the external input, respectively, and ξ_*i*_ is the white noise satisfying 〈ξ_*i*_〉 = 0 and $\langle \xi_{i}\left(t\right)\xi_{j}\left(t^{\prime }\right)\rangle =\delta_{ij}\delta \left(t-t^{\prime }\right)$. Every time when the membrane potential *V*_*i*_ crosses the threshold *V*_th_ = −40 mV, neuron *i* would emit a spike and *V*_*i*_ would be reset to *V*_r_.

The network consists of *N*_E_ excitatory neurons and *N*_I_ inhibitory neurons. We use *N*_E_ = 250, *N*_I_ = 250. The connections between these neurons are of the all-to-all type. We keep the inhibitory-to-excitatory (EI), excitatory-to-inhibitory (IE) and inhibitory-to-inhibitory (II) synaptic strengths constant during the entire simulation while the excitatory-to-excitatory (EE) connections are subject to the STDP learning rule to be introduced in section “The effect of firing rate and firing variability on learning dynamics—numerical evidence”. At the beginning of simulations, the strengths for the EE and IE are drawn at random from a uniform distribution from 0 mV to $w_{\text{EE}}^{\text{max}}=1$ mV and $w_{\text{IE}}^{\text{max}}=2$ mV, respectively, and the strengths for the EI and II connections are drawn at random from a uniform distribution from $w_{\text{EI}}^{\text{max}}=-4$ mV and $w_{\text{II}}^{\text{max}}=-4$ to 0 mV, respectively.

Based on the mean and variance of external inputs, we divide the excitatory neurons into three populations in which the αth (α = 1, 2, 3) population receives the external input of the mean $\mu_{\text{ext},\alpha }^{\text{p}}$ and standard deviation $\sigma_{\text{ext},\alpha }^{\text{p}}$. The number of neurons in the αth (α = 1, 2, 3) population is denoted as *N*_α_.

### Statistical properties of a single LIF neuron

We first describe the statistical properties of a single LIF neuron. The dynamics of a single LIF neuron with finite synaptic time constant is governed by Equation (1) and the following equation:

(3) $$\begin{array}{r} \tau_{\text{s}}\frac{dI}{dt}=-I+\mu +\sigma \sqrt{\tau_{\text{m}}}\xi \left(t\right), \end{array}$$

where μ and σ are the mean and standard deviation of the external input respectively, and ξ(*t*) is the Gaussian white noise satisfying 〈ξ〉 = 0 and 〈ξ(*t*)ξ(*t*′)〉 = δ(*t* − *t*′).

When τ_s_ is much smaller than τ_m_, the mean firing rate $\bar{r}$ has the following approximation:

(4) $$\bar{r}=F(\mu ,\sigma )={\left[\tau_{\text{m}}\sqrt{\pi }{\int^{\text{​}}}_{-\frac{\mu }{\sigma }+\beta }^{\frac{V_{\text{th}}-V_{r}-\mu }{\sigma }+\beta }e^{x^{2}}(1+\text{erf}(x))dx\right]}^{-1},$$

where $\beta =-\varsigma \left(1/2\right)\sqrt{\tau_{\text{s}}/\left(2\tau_{\text{m}}\right)}$, erf(·) is the error function and ζ(·) is the Riemann-Zeta function (Fourcaud and Brunel, 2002).

We use a response kernel to characterize how the LIF neuron ensemble responds to a perturbation. Specifically, we consider Equation (1) and the following equation:

(5) $$\begin{array}{r} \tau_{\text{s}}\frac{dI}{dt}=-I+\mu +\sigma \sqrt{\tau_{\text{m}}}\xi \left(t\right)+u\left(t\right), \end{array}$$

where *u*(*t*) is a perturbation. When *u*(*t*) is weak, the difference between the instantaneous firing rate *r*(*t*) of neuronal ensemble that is governed by Equations (1, 5) and the mean firing rate $\bar{r}$ of neuronal ensemble that is governed by Equations (1, 3) is small. Therefore, we can approximate the instantaneous firing rate *r*(*t*) by the mean firing rate $\bar{r}$ plus a linear response term with respect to *u*(*t*), i.e.,

(6) $$\begin{array}{r} r\left(t\right)=\bar{r}+\left(h*u\right)\left(t\right), \end{array}$$

where *h*(τ) is the linear response kernel of Equations (1, 3) and the symbol ^*^ stands for temporal convolution.

The analytical results for the linear response kernel are available for the case of zero synaptic time constant (Fourcaud and Brunel, 2002). Recently, for the case of nonzero synaptic time constant, the analytical result was derived (Schuecker et al., 2015). Here, we use the reverse correlation method to compute it (Dayan and Abbott, 2001; Ostojic et al., 2009). Specifically, we inject a weak Gaussian white noise signal ξ_*s*_(*t*) with correlation $\langle \xi_{s}\left(t+\tau \right)\xi_{s}\left(t\right)\rangle =\sigma_{\text{s}}^{2}\delta \left(\tau \right)$ to the LIF neuron, i.e., adding ξ_*s*_(*t*) on the right hand side of Equation (3). Note that this testing white noise signal ξ_*s*_(*t*) is different from the external noise input ξ(*t*) in Equation (3). By using the spike-triggered average technique, we calculate the response kernel in the following way:

(7) $$\begin{array}{r} h\left(\tau \right)=\frac{\frac{1}{N_{T}}\sum_{i=1}^{N_{T}}\xi_{s}\left(t_{i}-\tau \right)\bar{r}}{\sigma_{\text{s}}^{2}}, \end{array}$$

where ${\left\{t_{i}\right\}}_{i=1}^{N_{T}}$ is the spike timing of the LIF neuron in the duration *T* and the firing rate $\bar{r}$ is given by Equation (4) (Dayan and Abbott, 2001). In our simulation, σ_s_ = 2 mV and *T* = 10^6^ ms. We use one realization of ξ_*s*_(*t*) and perform the average over 1,000 realizations of external noise input ξ(*t*).

### Linear response theory

Here, we describe how we obtain ${\bar{r}}_{i}$ and *h*_*i*_(τ) for *i* = 1, ⋯, *N* in a self-consistent manner for the network described in Equations (1, 2). By splitting the spike train *s*_*j*_(*t*) in Equation (2) into the mean firing rate and its fluctuations, we can rewrite Equation (2) in the following form:

(8) $$\begin{array}{l} \frac{dI_{i}}{dt}=-\frac{I_{i}}{\tau_{\text{s}}}+\overset{N}{\underset{j=1}{\sum }}w_{ij}{\bar{r}}_{j}+\frac{\mu_{\text{ext},i}}{\tau_{\text{s}}}+\frac{\sigma_{\text{ext},i}}{\tau_{\text{s}}}\sqrt{\tau_{\text{m}}}\xi_{i}\left(t\right)\\ +\;\overset{N}{\underset{j=1}{\sum }}w_{ij}\left(s_{j}-{\bar{r}}_{j}\right), \end{array}$$

where the term $\overset{N}{\underset{j=1}{\sum }}w_{ij}\left(s_{j}-{\bar{r}}_{j}\right)$ can be regarded as a perturbation. By enforcing the first-order Volterra approximation of Equations (1, 8), we arrive at Equation (31), where ${\bar{r}}_{i}$ and *h*_*i*_(τ) are the mean firing rate and response kernel, respectively, of Equation (1) and the following equation:

(9) $$\begin{array}{r} \frac{dI_{i}}{dt}=-\frac{I_{i}}{\tau_{\text{s}}}+\overset{N}{\underset{j=1}{\sum }}w_{ij}{\bar{r}}_{j}+\frac{\mu_{\text{ext},i}}{\tau_{\text{s}}}+\frac{\sigma_{\text{ext},i}}{\tau_{\text{s}}}\sqrt{\tau_{\text{m}}}\xi_{i}\left(t\right). \end{array}$$

Here, we can observe that, even with the same mean and variance value of external inputs, different neurons may still have different mean firing rates due to the randomness of synaptic strengths. To simplify the self-consistent derivation of mean firing rate, we make a further assumption that the mean firing rates of single neurons within the same population are approximated by the population-averaged mean firing rate. Denote the population-averaged mean firing rates of the different populations as ${\bar{r}}_{\alpha }^{\text{p}}$, α = 1, 2, 3, I, where I stands for the inhibitory population. Then, ${\bar{r}}_{\alpha }^{\text{p}}$ is the mean firing rate of the following equations:

(10) $$\begin{array}{r} \tau_{\text{m}}\frac{dV_{\alpha }}{dt}=\left(V_{r}-V_{\alpha }\right)+I_{\alpha },\text{ for }\;\alpha =1,2,3,\text{I}, \end{array}$$

(11) $$\begin{array}{r} \frac{dI_{\alpha }}{dt}=-\frac{I_{\alpha }}{\tau_{\text{s}}}+\left(\overset{3}{\underset{\beta =1}{\sum }}N_{\beta }w_{\alpha \beta }^{\text{p}}{\bar{r}}_{\beta }^{\text{p}}+N_{\text{I}}w_{\alpha \text{I}}^{\text{p}}{\bar{r}}_{\text{I}}^{\text{p}}\right)+\frac{\mu_{\text{ext},\alpha }^{\text{p}}}{\tau_{\text{s}}}\\ +\;\frac{\sigma_{\text{ext},\alpha }^{\text{p}}}{\tau_{\text{s}}}\sqrt{\tau_{\text{m}}}\xi_{\alpha }\left(t\right), \end{array}$$

where $w_{\alpha \beta }^{\text{p}}$ stands for the average synaptic strength from Population β to Population α. Therefore, ${\bar{r}}_{\alpha }^{\text{p}}$ can be determined in the following self-consistent way:

(12) $$\begin{array}{r} {\bar{r}}_{\alpha }^{\text{p}}=F^{-1}\left(\mu_{\alpha }^{\text{p}},\sigma_{\text{ext},\alpha }^{\text{p}}\right),\text{ for }\;\alpha =1,2,3,\text{I}, \end{array}$$

(13) $$\begin{array}{r} \mu_{\alpha }^{\text{p}}=\tau_{\text{s}}(\overset{3}{\underset{\beta =1}{\sum }}N_{\beta }w_{\alpha \beta }^{\text{p}}{\bar{r}}_{\beta }^{\text{p}}+N_{\text{I}}w_{\alpha \text{I}}^{\text{p}}{\bar{r}}_{\text{I}}^{\text{p}})+\mu_{\text{ext},\alpha }^{\text{p}}. \end{array}$$

### The power spectrum for the Hawkes process

The first derivation of the power spectrum of Hawkes process (i.e., Equation 31) was carried out by Hawkes (1971a), in which the Wiener-Hopf theory was used. Here, we follow the method in Grytskyy et al. (2013). First, for the spike correlation function **C**(τ), the following equations hold:

(14a) $$C(\tau )\equiv \langle s(t+\tau )s^{T}(t)\rangle -\langle s(t)\rangle \langle s^{T}(t)$$

(14b) $$\begin{array}{r} =\langle \left(\text{r}\left(t+\tau \right)-\bar{\text{r}}\right){\text{s}}^{T}\left(t\right)\rangle +\text{D}\delta \left(\tau \right) \end{array}$$

(14c) $$\begin{array}{r} =\langle \left[\text{h}*\text{w}\left(\text{s}-\bar{\text{r}}\right)\right]\left(t+\tau \right){\text{s}}^{T}\left(t\right)\rangle +\text{D}\delta \left(\tau \right) \end{array}$$

(14d) $$\begin{array}{r} =\left(\text{h}*\text{w}\text{C}\right)\left(t\right)+\text{D}\delta \left(\tau \right), \end{array}$$

for τ ≥ 0, where **s**(*t*) and **r**(*t*) are the spike train and the instantaneous firing rate of Hawkes process, respectively, and $\text{D}=\text{diag}\left\{{\bar{r}}_{1},\cdots \;,{\bar{r}}_{N}\right\}$. In Equation (14b), we use the fact that 〈**s**(*t* + τ)**s**^*T*^(*t*)〉 = 〈**r**(*t* + τ)**s**^*T*^(*t*)〉 + **D**δ(τ). In Equation (14c), we insert the Hawkes process (Equation 31). In Equation (14d), we take advantage of the definition of spike correlation. Next, define **y**(*t*) = **r**_**y**_(*t*) + **x**(*t*), where

(15) $$\begin{array}{r} \;{\text{r}}_{\text{y}}\left(t\right)=\bar{\text{r}}+\left[\text{h}*\text{w}\left({\text{r}}_{\text{y}}-\bar{\text{r}}+\text{x}\right)\right]\left(t\right), \end{array}$$

and **x**(*t*) is the Gaussian white noise with correlation 〈**x**(*t* + τ)**x**(*t*)〉 = **D**_**x**_δ(τ). For **y**(*t*),

(16a) $$\begin{array}{rcl} {\text{C}}_{\text{y}}\left(\tau \right) & \equiv & \langle \text{y}\left(t+\tau \right)\left({\text{y}}^{T}\left(t\right)-{\bar{\text{r}}}^{T}\right)\rangle \end{array}$$

(16b) $$\begin{array}{r} =\langle \left[\text{h}*\text{w}\left(\text{y}-\bar{\text{r}}\right)+\text{x}\right]\left(t+\tau \right)\left({\text{y}}^{T}\left(t\right)-\bar{\text{r}}\right)\rangle \end{array}$$

$$\begin{array}{r} =\left(\text{h}*\text{w}{\text{C}}_{\text{y}}\right)\left(\tau \right)+\langle \text{x}\left(t+\tau \right)\left({\text{r}}_{\text{y}}^{T}\left(t\right)-{\bar{\text{r}}}^{T}\right)\rangle \end{array}$$

(16c) $$\begin{array}{r} +\;\langle \text{x}\left(t+\tau \right){\text{x}}^{T}\left(t\right)\rangle , \end{array}$$

(16d) $$\begin{array}{r} =\left(\text{h}*\text{w}{\text{C}}_{\text{y}}\right)\left(\tau \right)+{\text{D}}_{\text{x}}\delta \left(\tau \right), \end{array}$$

for τ ≥ 0. In Equation (16b), we have made use of the definition of **y**(*t*). In Equation (16c), we take advantage of the definition of **C**_**y**_(*t*). In Equation (18), we use the fact that $\langle \text{x}\left(t+\tau \right){\text{r}}_{\text{y}}^{T}\left(t\right)\rangle =\langle \text{x}\left(t+\tau \right)\rangle \langle {\text{r}}_{\text{y}}^{T}\left(t\right)\rangle =0$. By setting **D**_**x**_ = **D**, we obtain **C**(τ) = **C**_**y**_(τ). Therefore, to understand the correlation structure of the Hawkes process, it suffices to study the correlation structure of **y**(*t*). Define ${\hat{\text{r}}}_{\text{y}}\left(\omega \right)$ as the Fourier transform of **r**_**y**_(*t*). From Equation (16b), we have

(17) $$\begin{array}{r} {\hat{\text{r}}}_{\text{y}}=\hat{\text{h}}\left(\omega \right)\text{w}\left({\hat{\text{r}}}_{\text{y}}+\hat{\text{x}}\right). \end{array}$$

Thus, $\hat{\text{y}}={\hat{\text{r}}}_{\text{y}}+\hat{\text{x}}={\left(1-\hat{\text{h}}\text{w}\right)}^{-1}\hat{\text{x}}$. By using the Wiener-Khinchin theorem, we have

(18) $$\begin{array}{l} \hat{\text{C}}\left(\omega \right)=\langle \hat{\text{y}}\left(\omega \right){\hat{\text{y}}}^{T}\left(-\omega \right)\rangle \\ ={\left(1-\hat{\text{h}}\left(\omega \right)\text{w}\right)}^{-1}\text{D}{\left(1-{\text{w}}^{T}\hat{\text{h}}\left(-\omega \right)\right)}^{-1}. \end{array}$$

This completes the derivation of the power spectrum formula as discussed in Equation (32).

### Population-averaged spike correlation

Assume that there are *N*_α_ (α = 1, 2, 3, *I*) neurons in the αth population. The spike correlation between population α and population β is defined as follows:

(19) $$\begin{array}{r} \;C_{\alpha \beta }^{\text{p}}\left(\tau \right)=\frac{1}{N_{\alpha }N_{\beta }}\underset{i\in \alpha ,j\in \beta }{\sum }C_{ij}\left(\tau \right). \end{array}$$

The corresponding spike cross correlation is defined as follows:

(20) $$\begin{array}{r} \;C_{\alpha \beta }^{\text{p},\text{cr}}\left(\tau \right)=\frac{1}{N_{\alpha }N_{\beta }}\underset{i\in \alpha ,j\in \beta ,i\neq j}{\sum }C_{ij}\left(\tau \right). \end{array}$$

Note that $C_{\alpha \beta }^{\text{p},\text{cr}}\left(\tau \right)$ is used in the caption of Figure 4. Inserting Equation (16a) into Equation (19), we can obtain

(21) $$\begin{array}{l} C_{\alpha \beta }^{\text{p}}(\tau )=\frac{1}{N_{\alpha }N_{\beta }}\{\sum_{\gamma }\sum_{i\in \alpha ,j\in \beta ,k\in \gamma }[h_{i}*w_{ik}C_{kj}](\tau )\\ \;+\;\sum_{i\in \alpha ,j\in \beta }D_{i}\delta_{ij}\delta (\tau )\}\\ \;=\sum_{\gamma }h_{\alpha }^{\text{p}}w_{\alpha \gamma }^{\text{p}}N_{\gamma }C_{\gamma \beta }(\tau )+\frac{{\bar{r}}_{\alpha }^{\text{p}}}{N_{\alpha }}\delta_{\alpha \beta }\delta (\tau ), \end{array}$$

where $h_{\alpha }^{\text{p}}\left(\tau \right)$ stands for the linear response kernel of Population α. Similar to the result (Equation 18), the population-averaged power spectrum ${\hat{\text{C}}}^{\text{p}}\left(\cdot \right)={(\hat{C}}_{\alpha \beta }^{\text{p}}\left(\cdot )\right)$ has the following expression:

(22) $$\begin{array}{r} {\hat{\text{C}}}^{\text{p}}\left(\omega \right)={\left(1-{\hat{\text{h}}}^{\text{p}}\left(\omega \right){\text{w}}^{\text{p}}\right)}^{-1}{\text{D}}^{\text{p}}{\left(1-{{\text{w}}^{\text{p}}}^{T}{\hat{\text{h}}}^{\text{p}}\left(-\omega \right)\right)}^{-1}, \end{array}$$

where ${\text{h}}^{\text{p}}\left(\cdot \right)=\text{diag}\left\{h_{1}^{\text{p}}\left(\cdot \right),h_{2}^{\text{p}}\left(\cdot \right),h_{3}^{\text{p}}\left(\cdot \right),h_{I}^{\text{p}}\left(\cdot \right)\right\}$, ${\text{w}}^{\text{p}}={(w}_{\alpha \beta }^{\text{p}})$ and ${\text{D}}^{\text{p}}=\text{diag}\left\{{\bar{r}}_{1}^{\text{p}}/N_{1},{\bar{r}}_{2}^{\text{p}}/N_{2},{\bar{r}}_{3}^{\text{p}}/N_{3},{\bar{r}}_{I}^{\text{p}}/N_{I}\right\}$.

### Decomposition of cross correlation structures

Formally, the power spectrum (Equation 18) can be expanded in the following way:

(23) $$\begin{array}{r} \hat{\text{C}}\left(\omega \right)=\text{D}+\hat{\text{h}}\left(\omega \right)\text{w}\text{D}+\text{D}{\text{w}}^{T}{\hat{\text{h}}}^{T}\left(-\omega \right)+\hat{\text{h}}\left(\omega \right)\text{w}\text{D}{\text{w}}^{T}{\hat{\text{h}}}^{T}\left(-\omega \right)+\cdots \;. \end{array}$$

Therefore, the cross correlation **C**(τ), which is the inverse Fourier transform of $\hat{\text{C}}\left(\omega \right)$ by the Wiener-Khinchin theorem, can be approximated by Equation (33). The same decomposition can also be carried out for the population-averaged cross correlation as discussed previously.

## Results

**Figure 1:**
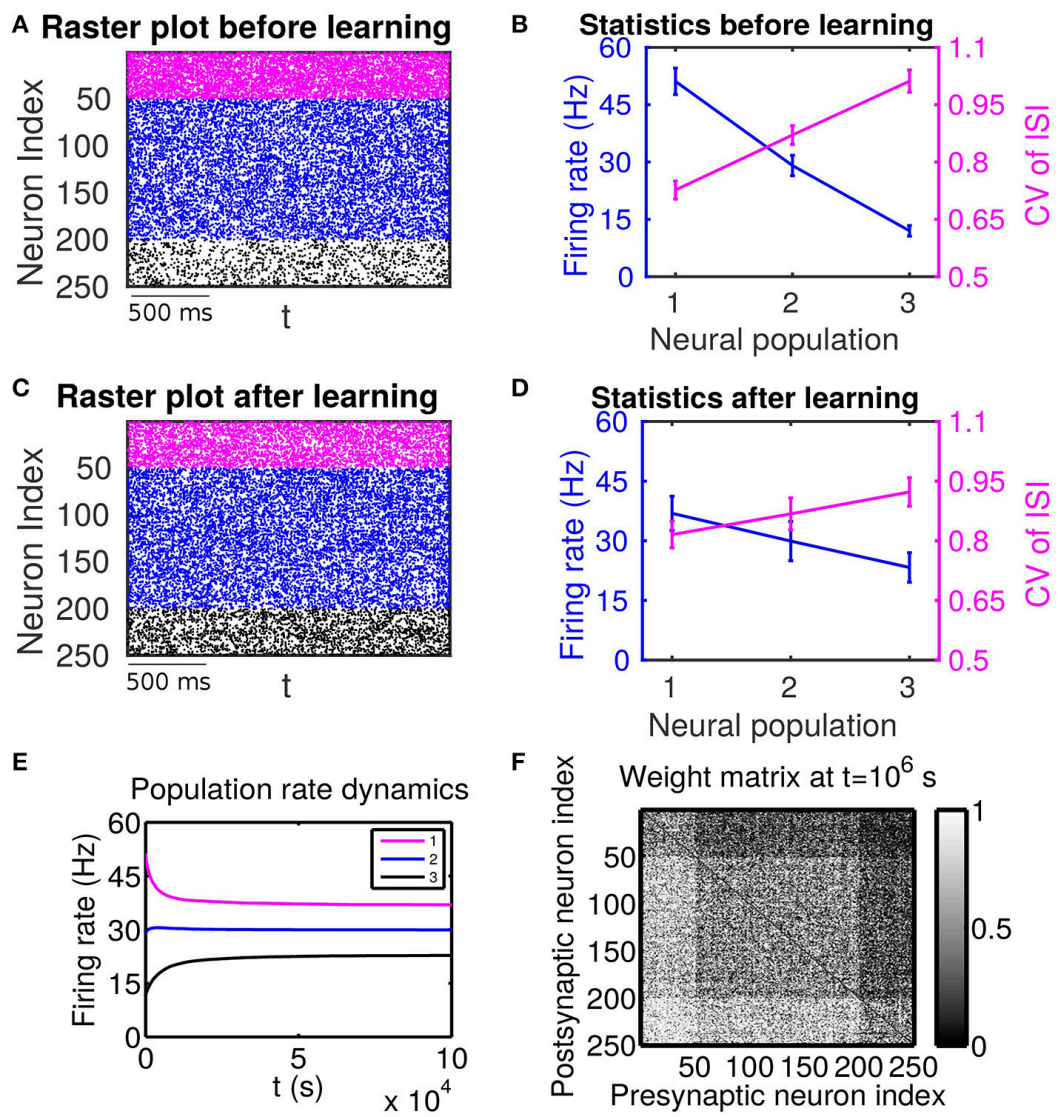
The effect of firing rate and variability on STDP learning dynamics—Case I. All excitatory neurons receive external inputs with an identical variance (i.e., $\sigma_{\text{ext},\alpha }^{\text{p}}=15.8$ mV for α = 1, 2, 3). Based on the values of mean of external inputs, the excitatory neurons are divided into three populations, in which Population 1 (including neurons labeled by 1–50, purple), Population 2 (including neurons labeled by 51–200, blue) and Population 3 (including neurons labeled by 201–250, black) receive the largest ($\mu_{\text{ext},1}^{\text{p}}=40$ mV), intermediate ($\mu_{\text{ext},2}^{\text{p}}=30$ mV) and smallest ($\mu_{\text{ext},3}^{\text{p}}=20$ mV) mean, respectively. The initial values of excitatory-to-excitatory connection strengths are drawn from a uniform distribution ranging from 0 to 1 mV. **(A,C)** Raster plots before learning and after learning, respectively. **(B,D)** Firing rates and coefficients of variation (CVs) of the different populations before learning and after 10^6^ s of learning, respectively. Error bars indicate the standard deviation of corresponding values within each population. **(E)** Firing rate dynamics of the different populations during learning. **(F)** Weight matrix after 10^6^ s of learning. The largest (i.e., brightest) and the smallest (i.e., darkest) value of the gray bar is 1 and 0 mV, respectively.

**Figure 2:**
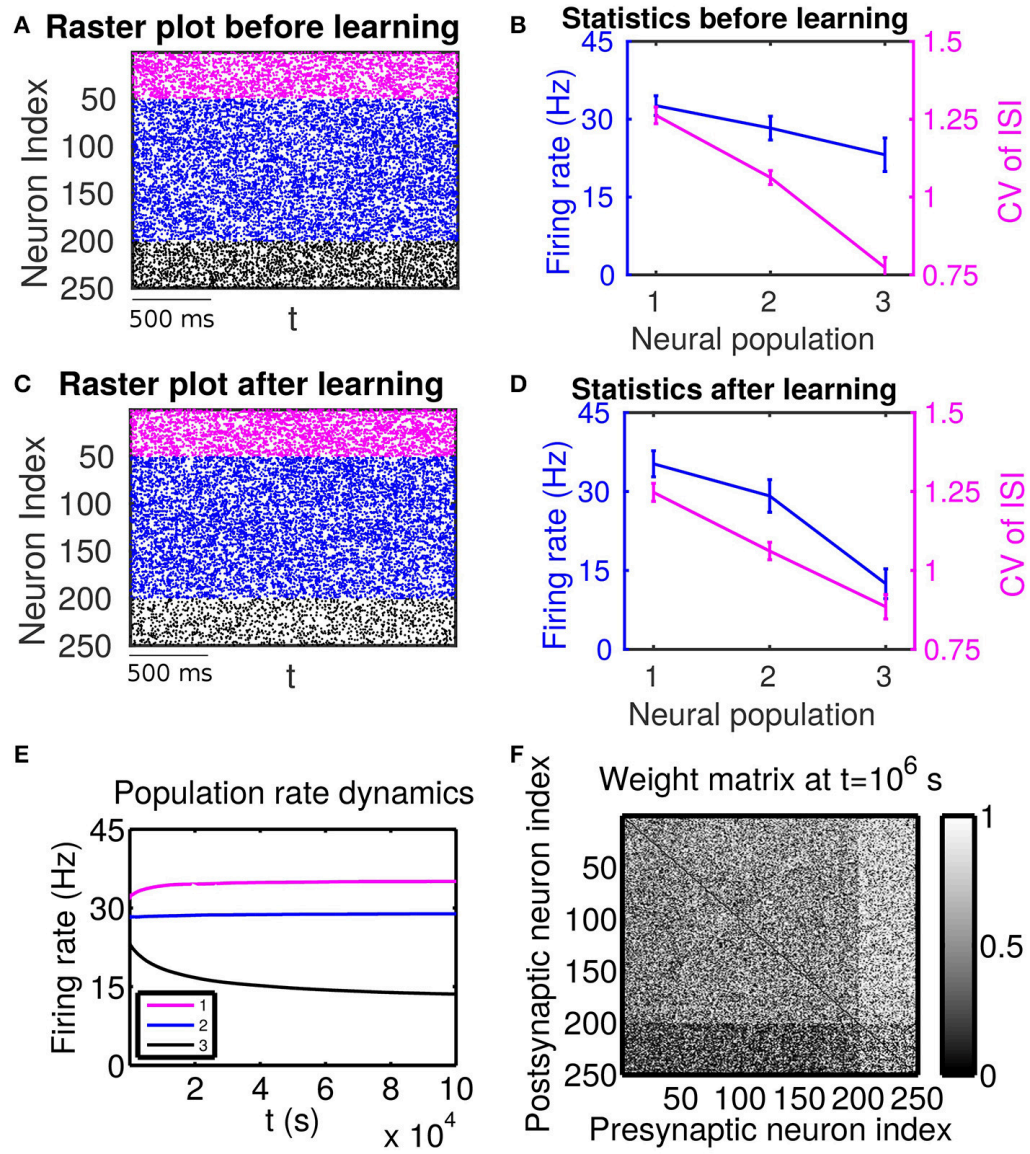
The effect of firing rate and variability on STDP learning dynamics—Case II. Different populations receive external inputs with different means as well as different variances. Population 1 (including neurons labeled by 1–50, purple) receives the smallest mean ($\mu_{\text{ext},1}^{\text{p}}=27.5$ mV) but the largest variance ($\sigma_{\text{ext},1}^{\text{p}}=31.6$ mV), Population 2 (including neurons labeled by 51–200, blue) receives an intermediate mean ($\mu_{\text{ext},2}^{\text{p}}=30$ mV) and variance ($\sigma_{\text{ext},2}^{\text{p}}=22.4$ mV), and Population 3 (including neurons labeled by 201–250, black) receives the largest mean ($\mu_{\text{ext},3}^{\text{p}}=32.5$ mV) but the smallest variance ($\sigma_{\text{ext},3}^{\text{p}}=11.2$ mV). The initial values of excitatory-to-excitatory connection strengths are drawn from a uniform distribution ranging from 0 to 1 mV. **(A,C)** Raster plots before learning and after learning, respectively. **(B,D)** Firing rates and coefficients of variation (CVs) of the different populations before learning and after 10^6^ s of learning, respectively. Error bars indicate the standard deviation of corresponding values within each population. **(E)** Firing rate dynamics of the different populations during learning. **(F)** Weight matrix after 10^6^ s of learning. The largest (i.e., brightest) and the smallest (i.e., darkest) value of the gray bar is 1 and 0 mV, respectively.

We first present the numerical evidence of the complexity of mutual interactions between plastic network structures and spiking activity, as it relates to the effect of high-order statistical structures implied in the STDP learning dynamics. In particular, we emphasize the importance of firing variability of spiking activity for the network structures learned through STDP. This fact is particularly emphasized by the sharp contrast of learned network structures between two scenarios (Case I in Figure 1 and Case II Figure 2); they share the same trend for firing rate change across different neuronal populations, but have the opposite trend for their firing variability change. We then develop a self-consistent linear response theory to account for the effects of both firing rate and firing variability on learning dynamics and explain the role observed in our numerical study of high-order statistics in learning dynamics.

### The effect of firing rate and firing variability on learning dynamics—numerical evidence

For our numerical experiments, we use the current-based leaky integrate-and-fire (LIF) neuron model with finite synaptic time constant (Babadi and Abbott, 2013). The network consists of 250 excitatory neurons and 250 inhibitory neurons (See section Methods for details). Based on the values of mean and variance of external inputs, we divide the excitatory neurons into three populations—Population 1 (denoted as P_1_, including neurons indexed from 1 to 50), Population 2 (denoted as P_2_, including neurons indexed from 51 to 200) and Population 3 (denoted as P_3_, including neurons indexed from 201 to 250). The initial values of excitatory-to-excitatory (EE) connection strengths are drawn at random from a uniform distribution ranging from 0 to $w_{\text{EE}}^{\text{max}}=1$ mV. During the simulation, the EE connections are subject to the canonical all-to-all pairwise additive STDP learning rule (Song et al., 2000; Babadi and Abbott, 2013). Specifically, let τ = *t*_*i*_ − *t*_*j*_, where *t*_*i*_ and *t*_*j*_ are a pair of spike times of excitatory neuron *i* and excitatory neuron *j*, respectively. This pair of spike times will affect a change of the synaptic strength *w*_*ij*_ from neuron *j* to neuron *i* as follows: *w*_*ij*_ → *w*_*ij*_ + *L*(τ), where the STDP learning window *L*(τ) is given by

(24) $$L(\tau )=\{\begin{array}{cc} A_{+}e^{-\tau /\tau_{+}}\; & \text{for​}\tau >0,\\ -A_{-}e^{-|\tau |/\tau_{-}}\; & \text{for​}\tau \leq 0. \end{array}$$

Similarly, for the synaptic strength *w*_*ji*_ from neuron *i* to neuron *j*, the update is *w*_*ji*_ → *w*_*ji*_ + *L*(−τ). As in Song et al. (2000) and Babadi and Abbott (2013), we enforce the hard bound to the value of *w*_*ij*_ and *w*_*ji*_, i.e., set *w*_*ij*_ (*w*_*ji*_) to $w_{\text{EE}}^{\text{max}}$ if $w_{ij}\left(w_{ji}\right)>w_{\text{EE}}^{\text{max}}$ and 0 if *w*_*ij*_(*w*_*ji*_) < 0. In this work, we will restrict ourselves to the balanced case in which *A*_+_ = *A*_−_ = 0.005 $w_{\text{EE}}^{\text{max}}$ and τ_+_ = τ_−_ = 20 ms. We characterize spiking activity in the network by its firing rate and coefficient of variation (CV), where CV is the ratio of the standard deviation of interspike interval (ISI) to the mean of ISI. As is well known, for a given neuron under fixed input conditions, CV is often used as a measure of firing variability — the larger CV, the more irregular the spiking activity.

If these populations receive external inputs of different means but an identical variance, we observe that, as the mean external input increases, the firing rate increases whereas the CV decreases. Figure 1B illustrates the corresponding firing statistics of these populations. For this case, as shown in Figure 1F, the connections from the populations of higher firing rate to those of lower firing rate are strengthened after learning whereas the connections with the opposite situation (i.e., connections from those of lower firing rate to those of higher firing rate) are weakened. This result is consistent with results of the previous studies (Morrison et al., 2007; Kozloski and Cecchi, 2010; Babadi and Abbott, 2013). Here, STDP is found to act as a firing rate equalizer, i.e., decreasing the rate of the neuronal populations of higher firing rate and increasing the rate of the neuronal populations of lower firing rate. Figure 1E displays an example of this behavior. Furthermore, by comparing the firing statistics before learning (Figure 1B) with that after learning (Figure 1D), we observe that STDP also acts as a firing variability equalizer, i.e., decreasing the CV of higher-CV neuronal populations and increasing the CV of lower-CV neuronal populations. For ease of discussion below, this case will be termed as Case I.

Next, we consider the case in which both the mean and variance of external inputs vary across these populations. We adjust both the mean and variance of external inputs (see the caption of Figure 2 for the specific values) such that Population 1 has the highest firing rate and the highest CV, Population 2 has an intermediate firing rate and CV, and Population 3 has the lowest firing rate and the lowest CV. The corresponding firing statistics of these populations before learning is displayed in Figure 2B. From Figure 2F, it can be seen clearly that after learning the connections from Population 1, which has the highest firing rate, to Population 3, which has the lowest firing rate, are weakened. We note that this phenomenon still exists even when the projections from the excitatory populations to the inhibitory populations become plastic (Supplementary Material). Furthermore, by comparing Figure 2B with Figure 2D, we observe that STDP tends to increase the rate of the population of the highest firing rate and decrease the rate of the population of the lowest firing rate. That is, STDP is no longer acting as a firing rate equalizer. However, it still acts as a weak equalizer for firing variability. This phenomenon cannot be explained solely by the firing rate theory (Morrison et al., 2007; Kozloski and Cecchi, 2010; Babadi and Abbott, 2013), in which the connections from the population of higher firing rate to the population of lower firing rate should be strengthened and STDP should act as a firing rate equalizer. In the following, we develop a linear response theory to characterize the importance of high-order firing statistics in learning dynamics as illustrated in Figure 2. For ease of discussion below, the case in Figure 2 will be termed as Case II.

### STDP learning dynamics

To characterize the learning dynamics, we consider two mutually-connected neurons in a recurrent network, say neuron *i* and neuron *j*. The STDP learning rule introduced in the last section can be simply written in the following weight dynamics:

(25) $$\begin{array}{r} \frac{dw_{ij}}{dt}=A_{+}x_{j}^{+}\left(t\right)s_{i}\left(t\right)-A_{-}x_{i}^{-}\left(t\right)s_{j}\left(t\right), \end{array}$$

(26) $$\begin{array}{r} \frac{dx_{j}^{+}}{dt}=-\frac{x_{j}^{+}}{\tau_{+}}+s_{j}\left(t\right), \end{array}$$

(27) $$\begin{array}{r} \frac{dx_{i}^{-}}{dt}=-\frac{x_{i}^{-}}{\tau_{-}}+s_{i}\left(t\right), \end{array}$$

where *s*_*i*_(*t*) and *s*_*j*_(*t*) respectively stand for the spike trains of neuron *i* and neuron *j*. Expressing $x_{j}^{+}$and $x_{i}^{-}$ as the integral of *s*_*j*_(*t*) and *s*_*i*_(*t*), respectively, we can obtain

(28) $$\begin{array}{r} \frac{dw_{ij}}{dt}=\int_{-\infty }^{\infty }L\left(\tau \right)s_{j}\left(t-\tau \right)s_{i}\left(t\right)d\tau . \end{array}$$

By assuming that the change of *w*_*ij*_(*t*) is sufficiently slow (Ocker et al., 2015), *s*_*j*_(*t* − τ)*s*_*i*_(*t*) can be approximated by *C*_*ij*_(τ) + 〈*s*_*i*_(*t*)〉〈*s*_*j*_(*t*)〉 where *C*_*ij*_(τ), defined as 〈*s*_*i*_(*t* + τ)*s*_*j*_(*t*)〉 − 〈*s*_*i*_(*t*)〉〈*s*_*j*_(*t*)〉, is the temporal spike cross correlation between neuron *i* and neuron *j* in the recurrent network with a fixed synaptic strength matrix. Combining the dynamics of *w*_*ji*_ in Equation (25), we arrive at the following equations:

(29) $$\begin{array}{r} \frac{dw_{ij}}{dt}=\int_{-\infty }^{\infty }L\left(\tau \right)C_{ij}\left(\tau \right)d\tau , \end{array}$$

(30) $$\begin{array}{r} \frac{dw_{ji}}{dt}=\int_{-\infty }^{\infty }L\left(\tau \right)C_{ji}\left(\tau \right)d\tau . \end{array}$$

Note that unlike the scenario studied in Babadi and Abbott (2013) and Ocker et al. (2015), there is no offset term in Equations (29, 30). This is due to the balanced condition *A*_+_ = *A*_−_ and τ_+_ = τ_−_ in our STDP learning rule. Therefore, to understand the learning dynamics (Equations 29, 30), it is facilitative to study the correlation structure in recurrent networks with static couplings.

In the following sections, we will follow the linear response theory proposed in Pernice et al. (2012), Trousdale et al. (2012), and Helias et al. (2013) and develop a self-consistent framework for the learning dynamics (Equations 29, 30). To this end, we first characterize how the firing variability information is encoded in the linear response kernel for the single LIF neuron case in section Incorporation of Firing Variability in Linear Response Kernel. Then, in section Hawkes Process as an Approximation of an LIF Network Dynamics, we approximate the LIF network dynamics with a stochastic point process called Hawkes process in which the instantaneous firing rate depends on the spiking history of interacting neurons (Hawkes, 1971b). With this Hawkes process approximation, in section Spike Correlation Structure of Hawkes Process, we discuss the issue of how firing statistics of spiking activity and network connectivity structure shape the spike correlation structure of the LIF network dynamics. In the end, we combine the correlation structure analysis with the STDP learning dynamics (Equations 29, 30) to arrive at the final learning dynamics (Equations 35, 36), which explain the observed numerical results, in section Learning Dynamics with Spike Correlation Decomposition.

### Incorporation of firing variability in linear response kernel

**Figure 3:**
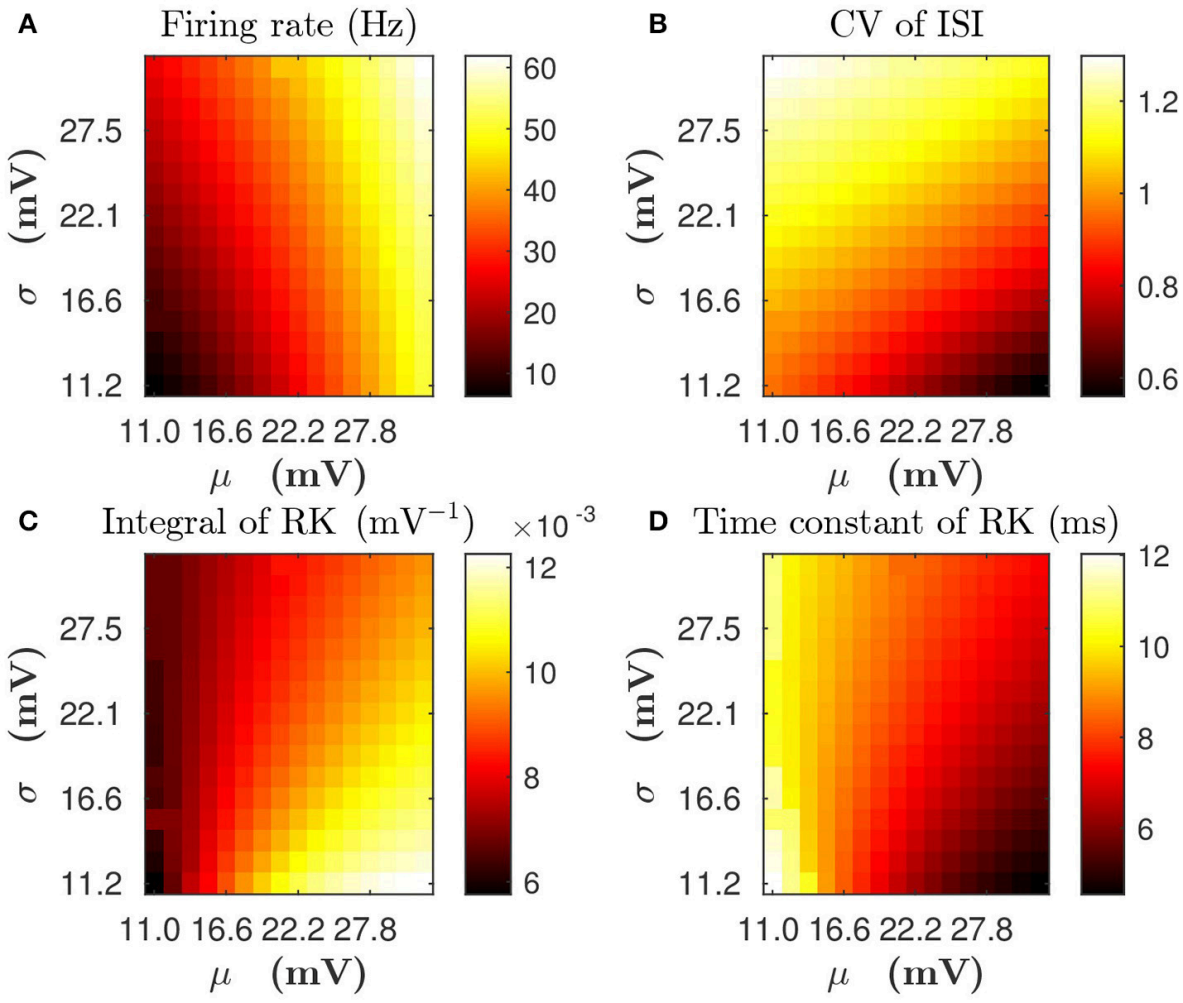
The dependence of statistical properties of the LIF neuron on the mean μ and the standard deviation σ of external inputs. **(A,B)** Firing rate and CV of inter-spike interval (ISI), respectively. **(C,D)** Integral of response kernel ∫*h*(τ)*dτ* and linear response time constant τ_eff_, respectively.

For a single LIF neuron described by Equations (1, 3), the dependence of mean firing rate and CV on input parameters (mean input value μ and standard deviation value σ) is illustrated in Figures 3A,B, respectively. By using the reverse correlation method (Dayan and Abbott, 2001; Ostojic et al., 2009) (see section Methods for details), We also computed the response kernel *h*(τ) that characterizes how the LIF neuron ensemble responds to a perturbation. We found that response kernel *h*(τ) exhibits an exponential decay profile, *A* exp{−τ/τ_eff_}Θ(τ), where Θ(τ) is the Heaviside function, provided that fluctuations of the external input are sufficiently high, i.e., comparable to the difference between firing threshold and reset voltage *V*_th_ − *V*_r_ (for example, σ is about 10 ~ 30 mV). We can use two quantities — the integral of response kernel ∫*h*(τ)*dτ*, which is τ_eff_*A* after the substitution of the exponential form of *h*(τ) = *A* exp{−τ/τ_eff_}Θ(τ), and the decay time constant τ_eff_ — to characterize *h*(τ). Figures 3C,D illustrate the dependence of ∫*h*(τ)*dτ* and τ_eff_ on μ and σ, respectively. In Figure 3, we can observe that, for any fixed mean firing rate, when the standard deviation σ increases, both CV of ISI and τ_eff_ increase whereas ∫*h*(τ)*dτ* decreases. Therefore, if there are two neurons with the same mean firing rate but different firing CVs, both the decay time constant and the integral of response kernel of these two neurons will differ from each other. In this sense, the information of firing variability is incorporated in the linear response kernel.

### Hawkes process as an approximation of an LIF network dynamics

In general, the response of a neuron to recurrent spike inputs is nonlinear. Here, we investigate the asynchronous state, as shown in Figures 1A, 2A, in which spikes from different neurons arrive at the synapses of the target neuron at different times. By considering that the magnitude of a postsynaptic potential elicited by the firing event of a single presynaptic neuron is in general small, i.e., the synaptic strength is weak, it is reasonable to treat the asynchronous recurrent inputs in a linear manner (Pernice et al., 2012; Trousdale et al., 2012; Helias et al., 2013). Specifically, by regarding $\overset{N}{\underset{j=1}{\sum }}w_{ij}\left(s_{j}-{\bar{r}}_{j}\right)$ in the LIF neuronal network as a perturbation, we arrive at the following effective description:

(31) $$\begin{array}{r} r_{i}\left(t\right)={\bar{r}}_{i}+\left[h_{i}*\overset{N}{\underset{j=1}{\sum }}w_{ij}\left(s_{j}-{\bar{r}}_{j}\right)\right]\left(t\right), \end{array}$$

where ${\bar{r}}_{i}$ and *h*_*i*_(τ) are the mean firing rate and response kernel of the *i*th neuron that is driven by both the external input and the mean-driven recurrent input (see section Methods for details), respectively, and *N* is the number of neurons in the network. Note that (i) the mean firing rate ${\bar{r}}_{i}$ is determined by the self-consistent Equations (12, 13); and (ii) the response kernel *h*_*i*_(τ) is computed based on the single LIF neuron Equations (1, 3) with the total mean input value $\mu_{i}^{\text{p}}$ in Equation (13) and the standard deviation value $\sigma_{\text{ext},i}^{\text{p}}$ in Equation (12). Equation (31) can be understood in the following way. On the one hand, given spike trains *s*_*j*_(*t*) for *j* = 1, ⋯, *N*, Equation (31) provides a way to evolve the instantaneous firing rate of the *i*th neuron *r*_*i*_(*t*). On the other hand, from Equation (31), the spike train *s*_*i*_(*t*) can be regarded as being generated by a jump process with the instantaneous firing rate *r*_*i*_(*t*). Therefore, we have obtained a self-consistent stochastic jump process that produces both the instantaneous firing rate *r*_*i*_(*t*) and the stochastic spike train *s*_*i*_(*t*). This stochastic process Equation (31) is a Hawkes process (Hawkes, 1971b), which has been extensively used in the neuroscience literature (Pernice et al., 2012; Trousdale et al., 2012; Helias et al., 2013). From the discussion above, such a Hawkes process can be viewed as the first-order (i.e., linear) Volterra approximation of the LIF network dynamics.

### Spike correlation structure of Hawkes process

For the Hawkes process (Equation 31), its spike correlation has an explicit analytical form (Hawkes, 1971a). Specifically, the power spectrum $\hat{\text{C}}\left(\omega \right)$, by the Wiener-Khinchin theorem, is the Fourier transform of spike correlation **C**(τ) = (*C*_*ij*_(τ)) and has the following form (Hawkes, 1971a) (see section Methods for a derivation):

(32) $$\hat{C}(\omega )={\left(1-\hat{h}(\omega )w\right)}^{-1}D{\left(1-w^{T}{\hat{h}}^{T}(-\omega )\right)}^{-1},$$

where $\hat{\text{h}}\left(\omega \right)$ is the Fourier transform of the response kernel **h**(τ) = diag{*h*_1_(τ), ⋯, *h*_*N*_(τ)}, **w** is the synaptic strength matrix, and $\text{D}=\text{diag}\left\{{\bar{r}}_{1},\cdots \;,{\bar{r}}_{N}\right\}$.

**Figure 4:**
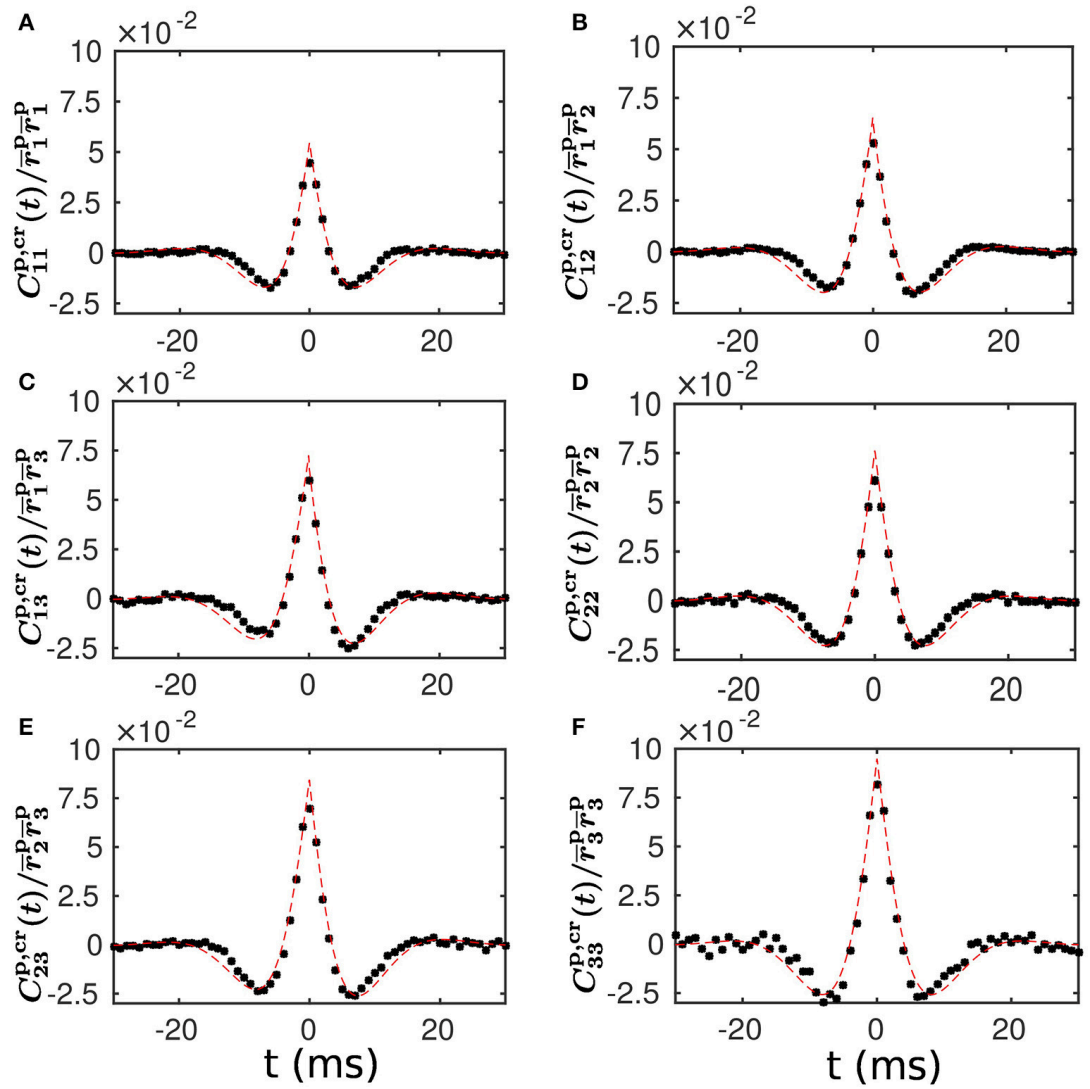
Population-averaged cross correlation—comparison between theoretical prediction and numerical results. **(A–F)** Normalized population-averaged cross correlation between the different populations. Note that $C_{\alpha \beta }^{\text{p},\text{cr}}$ for α, β = 1, 2, 3 is defined in Equation (20) and $r_{\alpha }^{\text{p}}$ for α = 1, 2, 3 is computed with Equations (12, 13) in a self-consistent way. The black dots are the cross correlation measured from a simulation of the LIF neuronal network with a static weight matrix. The red dashed line is the theoretical prediction of our linear response theory.

To examine the validity of our linear response theory, we compute the population-averaged cross correlation (see section Methods for details). We find that our theoretical prediction of the population-averaged cross correlation is in very good agreement with the cross correlation measured from our LIF neuronal network simulation. This agreement is clearly seen in Figure 4, demonstrating that our linear response theory can well capture the spike correlation structure of the LIF network dynamics.

We note that Equation (32) also provides a starting point to study how network connectivity affects the spike correlation structure since we can decompose the connections into direct connections, common inputs, and other higher-order connections. In particular, the spike correlation has the following decomposition (see section Methods for a derivation):

(33) $$\begin{array}{r} \text{C}\left(\tau \right)=\text{D}\delta \left(\tau \right)+\text{h}\left(\tau \right)\text{w}\text{D}+\text{D}{\text{w}}^{T}{\text{h}}_{-}^{T}\left(\tau \right)+\left(\text{h}\text{w}\text{D}{\text{w}}^{T}*{\text{h}}_{-}^{T}\right)\left(\tau \right)+\cdots \;, \end{array}$$

where **h**_−_(τ) ≡ **h**(−τ). Note that the similar decomposition has been derived in Pernice et al. (2012), Trousdale et al. (2012), Hu et al. (2013), and Ocker et al. (2015).

The first term on the right hand side (RHS) of Equation (33) can be obtained by setting the connection strength **w** in the Hawkes process (Equation 31) to zero. There is no cross correlation in this term.

For the second term on the RHS of Equation (33), the corresponding component with respect to the correlation between neuron *i* and neuron *j* is $h_{i}\left(\tau \right)w_{ij}{\bar{r}}_{j}$. This involves firing rate ${\bar{r}}_{j}$ of presynaptic neuron *j*, the connection strength *w*_*ij*_ from neuron *j* to neuron *i* and the linear response kernel *h*_*i*_(τ) of postsynaptic neuron *i*. Therefore, this component corresponds to the correlation induced by the direct connection from neuron *j* to neuron *i*. Similarly, the component $h_{j}\left(-\tau \right)w_{ji}{\bar{r}}_{i}$ (the third term on the RHS of Equation 33), corresponds to the correlation induced by the direct connection from neuron *i* to neuron *j*. Combining these two terms, we obtain the spike correlation induced by direct connections between neuron *i* and neuron *j*. For ease of discussion below, we denote these terms as $C_{ij}^{\text{di}}\left(t\right)$.

We next discuss the terms associated with the indirect connections. For the fourth term on the RHS of Equation (33), the corresponding component with respect to the correlation between neuron *i* and neuron *j* is $\underset{k}{\sum }w_{ik}{\bar{r}}_{k}w_{jk}\left[h_{i}*{h_{-}}_{j}\right]\left(\tau \right)$. This involves the firing rate ${\bar{r}}_{k}$ of neuron *k*, the connection strengths *w*_α*k*_ and the response kernels *h*_α_(·) for α = *i, j*. Therefore, this term corresponds to the correlation induced by the common inputs from neuron *k* to both neuron *i* and neuron *j*. As we have demonstrated above, *h*_α_(·), α = *i, j*, exhibits an exponential decay profile, which will be expressed as *B*_α_ exp(−*t*/τ_α_)Θ(*t*). Then, this component becomes

(34) $$\begin{array}{r} \underset{k}{\sum }B_{i}B_{j}\frac{\tau_{i}\tau_{j}}{\tau_{i}+\tau_{j}}w_{ik}{\bar{r}}_{k}w_{jk}\{\begin{array}{cc} \exp\left(-t/\tau_{i}\right)\; & \text{for }t\geq 0,\\ \exp\left(t/\tau_{j}\right)\; & \text{for }t<0. \end{array} \end{array}$$

For ease of discussion below, we denote this term as $C_{ij}^{\text{co}}\left(t\right)$, the remaining higher-order terms in Equation (33) as $C_{ij}^{\text{hi}}\left(t\right)$, and the summation of $C_{ij}^{\text{co}}\left(t\right)$ and $C_{ij}^{\text{hi}}\left(t\right)$ as the indirect term $C_{ij}^{\text{in}}\left(t\right)$. We can observe from Equation (34) that, if there is a difference between response time constants τ_*i*_ and τ_*j*_, $C_{ij}^{\text{co}}\left(t\right)$ will show an asymmetric property with respect to the axis *t* = 0. More precisely, if we assume τ_*i*_ > τ_*j*_, $C_{ij}^{\text{co}}\left(t\right)>C_{ij}^{\text{co}}\left(-t\right)$ for any *t* > 0. This asymmetry can be understood intuitively as follows. When there is a common input from neuron *k* into both neurons *i* and *j*, the neuron with the smaller response time constant (neuron *j*) will respond more rapidly than the neuron with the larger response time constant (neuron *i*) on average. Therefore, there is a higher likelihood that neuron *j* will fire before neuron *i*. In terms of the spike correlation, this implies that $C_{ij}^{\text{co}}\left(t\right)>C_{ji}^{\text{co}}\left(t\right)=C_{ij}^{\text{co}}\left(-t\right)$ for any *t* > 0. We note that, as shown in Equation (34), the common inputs from both excitatory and inhibitory neurons into neuron *i* and neuron *j* induce a positive spike correlation between neuron *i* and neuron *j*. Therefore, if there is a significant difference between τ_*i*_ and τ_*j*_, there will be a substantial asymmetry between the positive-*t* component of $C_{ij}^{\text{co}}\left(t\right)$ and the corresponding negative-*t* component. This will have a profound impact on the structure of *C*_*ij*_(*t*) as discussed below.

Now we study how different connectivity patterns affect the cross correlation structure between different neuronal populations for Cases I and II in Figures 1, 2. As an example, we pick neuron *i* from Population 3 and neuron *j* from Population 1. Note that, for Case I in Figure 1, neuron *i* (*j*) has a lower (higher) firing rate and a higher (lower) firing variability. Whereas, for Case II in Figure 2, neuron *i* (*j*) has a lower (higher) firing rate and a lower (higher) firing variability.

**Figure 5:**
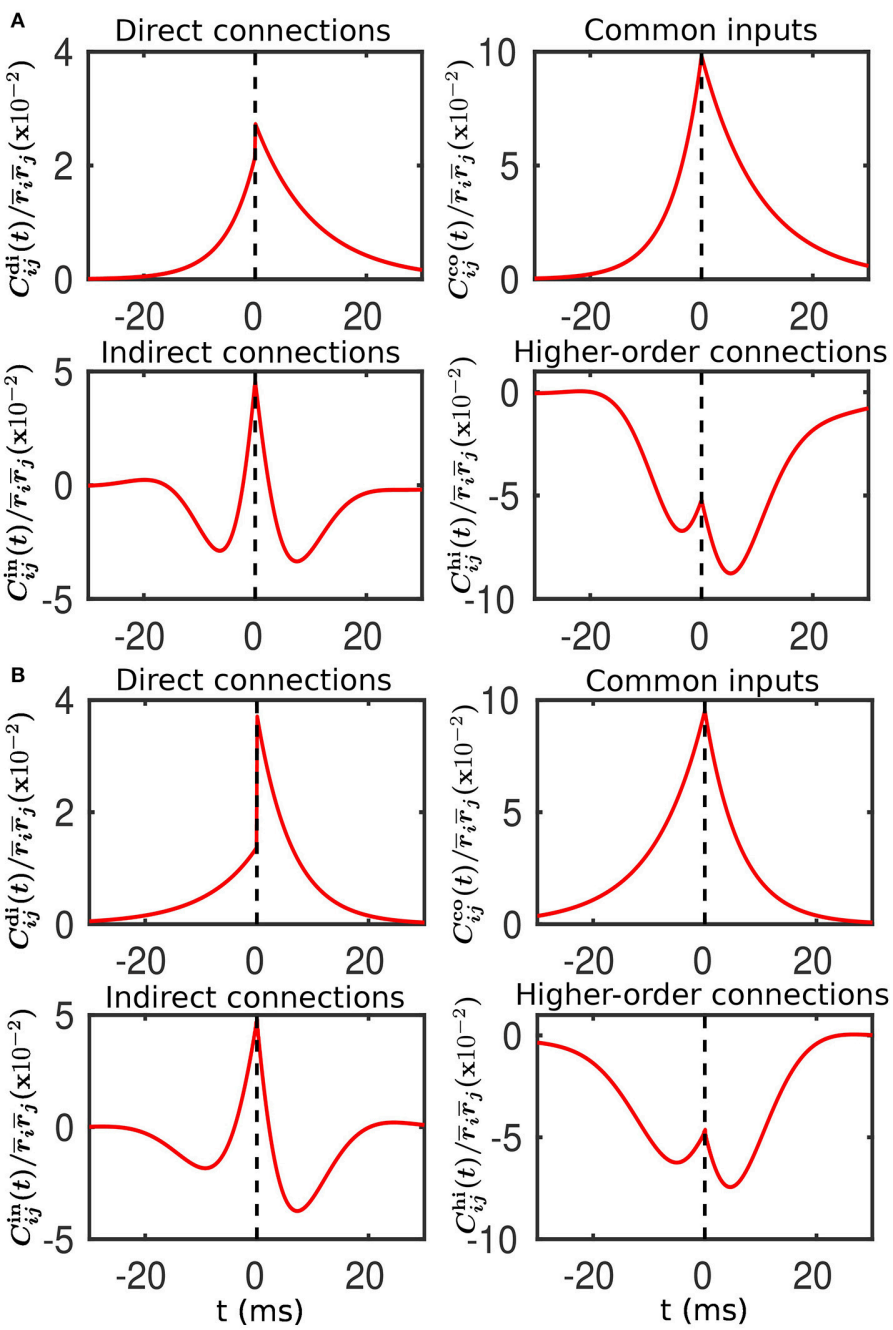
Decomposition of the spike cross correlation between neurons from the different populations before learning. Here, we choose neuron *i* from Population 3 and neuron *j* from Population 1. The connection strengths *w*_*ij*_ and *w*_*ji*_ are fixed at 0.5 mV. The top left, top right, bottom left and bottom right panels of both **(A,B)** correspond to the correlations associated with direct connections, common inputs, indirect connections, and higher-order connections, respectively. **(A)** Compared to neuron *i*, neuron *j* receives a larger mean and an identical variance of external inputs. This corresponds to Case I in Figure 1. **(B)** Compared to neuron *i*, neuron *j* receives a smaller mean but a larger variance of external inputs. This corresponds to Case II in Figure 2.

For Case I in Figure 1, we find that the net mean inputs to neuron *j* in Population 1 and neuron *i* in Population 3 are 31.45 and 11.55 mV, respectively. Note that the external mean inputs $\mu_{\text{ext},j}^{\text{p}}$ and $\mu_{\text{ext},i}^{\text{p}}$ are 40 and 20 mV, respectively. Since the net mean input is the summation of external mean input and recurrent input, the net recurrent inputs to neuron *j* and neuron *i* are −8.55 mV and −8.45 mV, respectively. This means the overall recurrent inputs to both neurons are inhibitory. In Case I, the standard deviation values for both neurons are 15.81 mV. With these net mean input values and standard deviation values, we compute the response kernel by using the reverse correlation method (see section Methods for more details). We find that ∫*h*_*j*_(*t*)*dt* > ∫*h*_*i*_(*t*)*dt* and τ_*j*_ < τ_*i*_, which is evident in Figures 3C,D. From the left panels of Figure 5A, we can observe that (i) the positive-*t* component of $C_{ij}^{\text{di}}\left(t\right)$ is larger than the corresponding negative-*t* component; and (ii) the positive-*t* component of $C_{ij}^{\text{in}}\left(t\right)$ is slightly smaller than the corresponding negative-*t* component. To further understand how different connectivity patterns shape $C_{ij}^{\text{in}}\left(t\right)$, we split $C_{ij}^{\text{in}}\left(t\right)$ into the common-input correlation $C_{ij}^{\text{co}}\left(t\right)$ and the higher-order-connection correlation $C_{ij}^{\text{hi}}\left(t\right)$. It can be seen clearly from the right panels of Figure 5A that the positive-*t* component of $C_{ij}^{\text{hi}}\left(t\right)$ is smaller than the corresponding negative-*t* component whereas the positive-*t* component of $C_{ij}^{\text{co}}\left(t\right)$ is larger than the corresponding negative-*t* component. A combination of these two facts yields a small difference between the positive-*t* component of $C_{ij}^{\text{in}}\left(t\right)$ and the corresponding negative-*t* component. We note that the asymmetry of $C_{ij}^{\text{co}}\left(t\right)$ with respect to the axis *t* = 0 exhibited in the top right panel of Figure 5A is a consequence of τ_*j*_ < τ_*i*_.

For Case II in Figure 2, we find that the net mean inputs to neuron *j* in Population 1 and neuron *i* in Population 3 are 15.58 and 20.50 mV, respectively. Note that the external mean inputs $\mu_{\text{ext},j}^{\text{p}}$ and $\mu_{\text{ext},i}^{\text{p}}$ are 27.5 and 32.5 mV, respectively. Since the net mean input is the summation of external mean input and recurrent input, the net recurrent inputs to neuron *j* and neuron *i* are −11.92 and −12.00 mV, respectively. This means the overall recurrent inputs to both neurons also are inhibitory. In Case II, the standard deviation values for neuron *j* and neuron *i* are 31.6 and 11.1 mV, respectively. With these net mean input values and standard deviation values, we compute the response kernel by using the reverse correlation method (see section Methods for more details). We find that ∫*h*_*j*_(*t*)*dt* < ∫*h*_*i*_(*t*)*dt* and τ_*j*_ > τ_*i*_, which is also evident in Figures 3C,D. From the left panels of Figure 5B, we can observe that (i) the positive-*t* component of $C_{ij}^{\text{di}}\left(t\right)$ is again larger than the corresponding negative-*t* component; and (ii) the positive-*t* component of $C_{ij}^{\text{in}}\left(t\right)$ is much smaller than the corresponding negative-*t* component. From the right panels of Figure 5B, we find that the positive-*t* components of both $C_{ij}^{\text{co}}\left(t\right)$ and $C_{ij}^{\text{hi}}\left(t\right)$ are smaller than the corresponding negative-*t* components. A combination of these two effects yields a large difference between the positive-*t* component of $C_{ij}^{\text{in}}\left(t\right)$ and the corresponding negative-*t* component. Note that the fact that $C_{ij}^{\text{co}}\left(t\right)<C_{ij}^{\text{co}}\left(-t\right)$ for *t* > 0 in Figure 5B is a consequence of the inequality τ_*j*_ > τ_*i*_. In the next section, we will demonstrate the extent of asymmetry between the positive-*t* component of $C_{ij}^{\text{in}}\left(t\right)$ and the corresponding negative-*t* component underlies the difference of network structures learned through STDP presented in Figures 1, 2.

### Learning dynamics with spike correlation decomposition

Combining the STDP learning dynamics (Equations 29, 30) with the direct- and indirect-connection decomposition of the correlation structure in the previous section, we arrive at the following learning dynamics:

(35) $$\begin{array}{r} \frac{dw_{ij}}{dt}=A_{i}w_{ij}{\bar{r}}_{j}-A_{j}w_{ji}{\bar{r}}_{i}+B, \end{array}$$

(36) $$\begin{array}{r} \frac{dw_{ji}}{dt}=A_{j}w_{ji}{\bar{r}}_{i}-A_{i}w_{ij}{\bar{r}}_{j}-B, \end{array}$$

where $A_{\alpha }=\int_{0}^{\infty }A_{+}\exp\left\{-\tau /\tau_{+}\right\}h_{\alpha }\left(\tau \right)d\tau$ for α = *i, j* and $B=\int L\left(\tau \right)C_{ij}^{\text{in}}\left(\tau \right)d\tau$. In Equations (35, 36), the first and second terms on the RHS correspond to the effect of direct connections between neuron *i* and neuron *j*, whereas the third term corresponds to the effect of indirect connections between neuron *i* and neuron *j*. Note that here we have used the balanced STDP assumption, i.e., *A*_+_ = *A*_−_ and τ_+_ = τ_−_.

**Figure 6:**
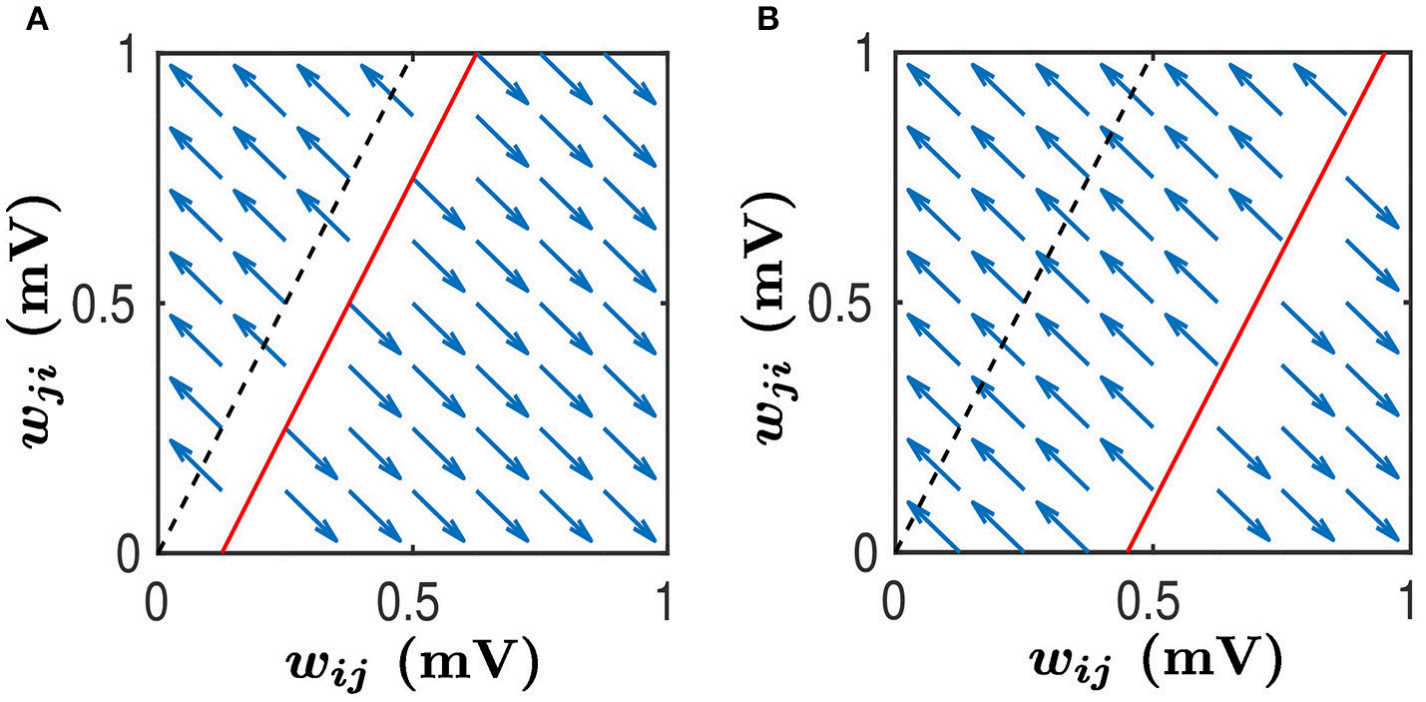
Phase plane analysis of learning dynamics. This learning dynamics has two attractors — (*w*_*ij*_, *w*_*ji*_) = (1, 0) and (*w*_*ij*_, *w*_*ji*_) = (0, 1). The black dashed line satisfies $A_{i}w_{ij}{\bar{r}}_{j}-A_{j}w_{ji}{\bar{r}}_{i}=0$ while the red line satisfies $A_{i}w_{ij}{\bar{r}}_{j}-A_{j}w_{ij}{\bar{r}}_{i}-B=0$. We assume that $A_{i}{\bar{r}}_{j}>A_{j}{\bar{r}}_{i}$. Therefore, the slope of both the black dashed line and the red line is greater than 1. Any (*w*_*ij*_, *w*_*ji*_) pair in the area above the red line will converge to (0, 1) whereas any pair in the area below the red line will converge to (1, 0), as indicated by the blue arrows. Therefore, the area above the red line is the basin of the attractor (0, 1) whereas the area below the red line is the basin of the attractor (1, 0). Note that *w*_*ij*_ and *w*_*ji*_ evolve along the line *w*_*ij*_ + *w*_*ji*_ = Const as indicated by the arrows. **(A)** The size of basin of the attractor (0, 1) is smaller than that of the attractor (1, 0). In this case, synapses from the neuronal population to which neuron *j* belongs, to the neuronal population to which neuron *i* belongs, would be more likely to be strengthened, whereas the synapses with the opposite direction will be weakened. **(B)** The size of basin of the attractor (0, 1) is larger than that of the attractor (1, 0). In this case, synapses from the neuronal population to which neuron *j* belongs, to the neuronal population to which neuron *i* belongs, would be more likely to be weakened, whereas the synapses with the opposite direction will be strengthened.

As argued in Babadi and Abbott (2013), while a linear system of differential equations such as Equations (35, 36) cannot have more than two attractors, the hard lower and upper bounds enforced by the STDP learning rule, i.e., the condition $0\leq w_{ij}\leq w_{\text{EE}}^{\text{max}}$, can lead to the following two attractors: $\left(w_{ij},w_{ji}\right)=\left(w_{\text{EE}}^{\text{max}},0\right)$ and $\left(w_{ij},w_{ji}\right)=\left(0,w_{\text{EE}}^{\text{max}}\right)$. For the sake of discussion, we consider $A_{i}{\bar{r}}_{j}>A_{j}{\bar{r}}_{i}$ below. Then, the line $w_{ji}=\frac{1}{A_{j}{\bar{r}}_{i}}\left(A_{i}{\bar{r}}_{j}w_{ij}+B\right)$ has the slope greater than 1, as illustrated by the black dash line in Figure 6. If neglecting the indirect connection effect, that is, setting *B* = 0, we can observe from Equations (35, 36) that, for any (*w*_*ij*_, *w*_*ji*_) pair satisfying $A_{i}w_{ij}{\bar{r}}_{j}-A_{j}w_{ji}{\bar{r}}_{i}>0$, the pair will converge to $\left(w_{\text{EE}}^{\text{max}},0\right)$. Whereas, for any (*w*_*ij*_, *w*_*ji*_) pair satisfying $A_{i}w_{ij}{\bar{r}}_{j}-A_{j}w_{ji}{\bar{r}}_{i}<0$, the pair will converge to $\left(0,w_{\text{EE}}^{\text{max}}\right)$. This is illustrated in Figure 6A with the black dash line separating the two basins. In this case, the size of basin of the attractor $\left(w_{\text{EE}}^{\text{max}},0\right)$ is larger than that of the attractor $\left(0,w_{\text{EE}}^{\text{max}}\right)$. Therefore, *w*_*ij*_ would be more likely to be potentiated, whereas *w*_*ji*_ would be more likely to be depressed.

When the indirect connection effect is included, there exist two distinct regimes depending on the value of *B*. For the sake of discussion, we denote the size of basin of $\left(w_{\text{EE}}^{\text{max}},0\right)$ (i.e., the area below the line $A_{i}w_{ij}{\bar{r}}_{j}-A_{j}w_{ji}{\bar{r}}_{i}+B=0$) as *S*_*ij*_ and the size of basin of $\left(0,w_{\text{EE}}^{\text{max}}\right)$ (i.e., the area above the line $A_{i}w_{ij}{\bar{r}}_{j}-A_{j}w_{ji}{\bar{r}}_{i}+B=0$) as *S*_*ji*_. One is a regime in which *S*_*ij*_ > *S*_*ji*_. This occurs when *B* ≥ 0 or *B* is negative but small. As illustrated in Figure 6A, the red line separates the two basins. In this regime, *w*_*ij*_ would be more likely to be potentiated whereas *w*_*ji*_ would be more likely to be depressed. For neuronal populations, the synapses from the neuron *j*'s population to neuron *i*'s population will have a higher likelihood to be strengthened. In contrast, the synapses with the opposite direction will be more likely to be weakened. The other is a regime in which *S*_*ij*_ < *S*_*ji*_. This occurs when *B* is sufficiently negative, as indicated by the red line in Figure 6B. In this regime, *w*_*ij*_ would be more likely to be depressed whereas *w*_*ji*_ would be more likely to be potentiated. For neuronal populations, the synapses from neuron *i*'s population to neuron *j*'s population will have a higher likelihood to be strengthened, whereas the synapses with the opposite direction will be weakened with a larger probability.

We now examine the cases in Figures 1, 2. For Case I in Figure 1 and Table 1 displays the corresponding values of $A_{i}{\bar{r}}_{j}$, $A_{j}{\bar{r}}_{i}$, *B*, *S*_*ij*_, *S*_*ji*_ as well as the average connection strengths ${\bar{w}}_{ij}$ and ${\bar{w}}_{ji}$ after 10^6^ s of learning. It can be seen clearly that for all the three (*i, j*) pairs, namely (*i* ∈ P_2_, *j* ∈ P_1_), (*i* ∈ P_3_, *j* ∈ P_1_) and (*i* ∈ P_3_, *j* ∈ P_2_), $S_{ij}>0.5{\text{mV}}^{2}>S_{ji}$, indicating that ${\bar{w}}_{ij}$ for all these three pairs would be strengthened. Indeed, this strengthening is confirmed by the values of ${\bar{w}}_{ij}$ presented in Table 1 (i.e., ${\bar{w}}_{ij}>0.5\text{mV}>{\bar{w}}_{ji}$ for all three pairs). To evaluate the contribution of common inputs and higher-order connections separately, we also include *B*^co^ (defined as $\int L\left(\tau \right)C_{ij}^{\text{co}}\left(\tau \right)d\tau$) and *B*^hi^ (defined as $\int L\left(\tau \right)C_{ij}^{\text{hi}}\left(\tau \right)d\tau$) in Table 1. We can observe that for all three pairs *B*^co^ > 0 whereas *B*^hi^ < 0. Since *B* = *B*^co^ + *B*^hi^, this gives rise to a negative but small value of *B*, yielding a regime in which *S*_*ij*_ > *S*_*ji*_. Since the connections from the populations of higher firing rate to those of lower firing rate are strengthened whereas the connections with the opposite direction are weakened, the rate of the populations of lower firing rate will increase whereas that of the populations of higher firing rate will decrease. The strengthening of connections from the populations of higher firing rate to those of lower firing rate leads to the increase of the mean of total inputs into the populations of lower firing rate. As illustrated in Figure 3B, when the variance of total inputs is fixed, the larger the mean total inputs, the smaller the CV. Therefore, this explains why in Figure 1 the CV of the populations of lower firing rate, which is larger before learning, decreases after learning. Therefore, STDP here acts as a weak equalizer for both firing rate and firing variability, as demonstrated in Figure 1.

**Table 1:** Parameters of learning dynamics (Equations 35, 36) and related quantities for Figure 1.

	**$A_{i}{\bar{r}}_{j}\times 10^{6}$ (ms^−1^)**	**$A_{i}{\bar{r}}_{j}\times 10^{6}$ (ms^−1^)**	***B* × 10^6^ (ms^−1^·mV)**	***B*^co^ × 10^6^ (ms^−1^·mV)**	***B*^hi^ × 10^6^ (ms^−1^·mV)**	***S*_*ij*_ (mV^2^)**	**${\bar{w}}_{ij}$ (mV)**	**${\bar{w}}_{ji}$ (mV)**
(*i* ∈ P_2_, *j* ∈ P_1_)	1.75	1.22	−0.19	0.47	−0.66	0.54	0.68	0.32
(*i* ∈ P_3_, *j* ∈ P_1_)	1.12	0.54	−0.18	0.80	−0.98	0.60	0.81	0.19
(*i* ∈ P_3_, *j* ∈ P_2_)	0.66	0.45	−0.04	0.44	−0.48	0.59	0.70	0.30

For Case II in Figure 2 and Table 2 shows that for all three (*i, j*) pairs, namely (*i* ∈ P_2_, *j* ∈ P_1_), (*i* ∈ P_3_, *j* ∈ P_1_) and (*i* ∈ P_3_, *j* ∈ P_2_), $S_{ij}<0.5\text{ m}{\text{V}}^{2}<S_{ji}$ and ${\bar{w}}_{ij}<0.5\text{ mV}<{\bar{w}}_{ji}$. Since the connections from the populations of higher firing rate to the populations of lower firing rate are weakened whereas the connections with the opposite direction are strengthened, the rate of the populations of higher firing rate will increase further whereas that of the populations of lower firing rate will decrease further. Meanwhile, the weakening of connections from the populations of higher firing rate to those of lower firing rate leads to the decrease of the mean of total inputs into the populations of lower firing rate. As illustrated in Figure 3B, the smaller the mean total inputs, the larger the CV. Therefore, the CV of these populations of lower firing rate, which is smaller before learning, increases after learning. Note that, in Case II, those populations of lower firing rate also have the lower firing variability. This explains why STDP acts as a weak equalizer of firing variability, instead of firing rate, in Figure 2.

**Table 2:** Parameters of learning dynamics (Equations 35, 36) and related quantities for Figure 2.

	**$A_{i}{\bar{r}}_{j}\times 10^{6}$ (ms^−1^)**	**$A_{i}{\bar{r}}_{j}\times 10^{6}$ (ms^−1^)**	***B* × 10^6^ (ms^−1^·mV)**	***B*^co^ × 10^6^ (ms^−1^·mV)**	***B*^hi^ × 10^6^ (ms^−1^ ·mV)**	***S*_*ij*_ (mV^2^)**	**${\bar{w}}_{ij}$ (mV)**	**${\bar{w}}_{ji}$ (mV)**
(*i* ∈ P_2_, *j* ∈ P_1_)	0.92	0.71	−0.15	−0.11	−0.04	0.45	0.45	0.55
(*i* ∈ P_3_, *j* ∈ P_1_)	1.28	0.63	−0.55	−0.53	−0.02	0.32	0.30	0.70
(*i* ∈ P_3_, *j* ∈ P_2_)	1.15	0.73	−0.40	−0.45	0.05	0.34	0.32	0.68

Furthermore, for Case II in Figure 2, by examining the contribution of common inputs and higher-order connections, we can find from Table 2 that, compared to *B*^co^, *B*^hi^ is negligible for the pairs (*i* ∈ P_2_, *j* ∈ P_1_) and (*i* ∈ P_3_, *j* ∈ P_1_). Therefore, for these two pairs, it is the contribution of common-input correlations that yields a large negative value of *B* and the corresponding network structures in Figure 2. Note that the asymmetric property of common-input correlations arises from the difference of response time constants between different neurons. As demonstrated in Figure 3, the response time constant encodes the information of the firing variability of spiking activity. In this sense, we have established a direct link between firing variability and learning dynamics by analyzing the spike correlation structures, and have demonstrated explicitly how firing variability shapes the network structures learned through STDP.

## Discussion

We have investigated how the high-order statistics of spiking activity affect the learning dynamics in a plastic LIF neuronal network. We have developed a self-consistent linear response theory, which incorporates the information of both firing rate and firing variability. By decomposing the spike correlation structure into the component associated with local direct connections and that associated with indirect connections, we have identified two distinct regimes regarding the network structures learned through STDP. In one regime, the contribution of the correlation associated with local direct connections dominates in STDP learning dynamics, leading to network structures consistent with the previous work (Morrison et al., 2007; Kozloski and Cecchi, 2010; Babadi and Abbott, 2013). In the other regime, the contribution of the correlation associated with indirect connections, instead of local direct connections, dominates, yielding network structures at variance with the prediction by the simple firing rate theory. The dominance of the indirect-connection correlation mainly arises from the asymmetric common-input correlations, which is induced by the heterogeneity of firing variability across different neuronal populations. Therefore, our study highlights the importance of firing variability of spiking activity for the evolution of plastic neural networks.

In a series of work (Gilson et al., 2009a,b,c,d, 2010), the STDP learning dynamics in recurrent networks has been investigated with Hawkes processes. However, the response kernel of the Hawkes process used there is identical across neuronal populations, thus the effect of firing variability was not addressed. Here, we study the STDP learning dynamics with the LIF neuron, in which the effect of firing variability is addressed by varying the mean and variance of external inputs across different neuronal populations. In Babadi and Abbott (2013), the authors developed a theory to explain the formation of in- and out-hubs. However, the effect of firing variability has not been discussed. Our theory can be viewed as an extension of the work (Babadi and Abbott, 2013). More recently, motif dynamics have been studied with linear response theory for one homogeneous population (Ocker et al., 2015). Instead, our work focuses on the evolution of connection strengths across different neuronal populations.

We have focused here on the balanced STDP rule. However, it is straightforward to extend our analysis to the unbalanced case by including additional terms in learning dynamics (Equations 35, 36). An unbalanced STDP rule can be expected to act as a loop-generating or loop-eliminating mechanism, depending on whether the rule is potentiation-dominated or depression-dominated (Babadi and Abbott, 2013). The neuron model we used here is the current-based LIF neuron. As we have demonstrated, the key property of a single neuron model to have an indirect-connection-dominated regime is that the firing variability of spiking activity can be encoded in the time constant of linear response kernel. Therefore, it can be expected that the indirect-connection-dominated regime can exist with other neuron models, such as conductance-based models, exponential integrate-and-fire models or more realistic Hodgkin-Huxley neurons. The connectivity here is of the all-to-all type. We can readily extend our self-consistent linear response theory to the sparsely connected network case. For larger network size, such as the network of 500 excitatory neurons and 500 inhibitory neurons, we have also found that there is an indirect-connection-dominated regime, provided that there is a significant difference of firing variability between different neuronal populations. In this work, the input statistics (i.e., input means and input variances) are kept constant, indicating that the circuit we studied receives the same sensory stimuli during the whole simulation. Under this assumption, the neural population activity can be simply quantified by mean firing rate and CV of ISI. It would be very interesting to study the learning dynamics when the stimulation periods are interleaved by spontaneous states. Another interesting issue to be addressed in the future work is how the high-order firing statistics of spiking activity affect the connectivity structures in a circuit with more complex learning rules, such as triplet STDP learning rule (Gjorgjieva et al., 2011).

In conclusion, we have demonstrated that the introduction of heterogeneity of firing variability across different neuronal populations can give rise to counterintuitive network structures in a plastic neural circuit. Our work suggests that the high-order statistics of spiking activity can play an important role in shaping the connectivity patterns in our brain.

## Author contributions

Conceived and designed the research, performed experiments, analyzed data, and wrote the paper: BM, DZ, and DC.

### Conflict of interest statement

The authors declare that the research was conducted in the absence of any commercial or financial relationships that could be construed as a potential conflict of interest.
