# Supplementary material for: Effects of Firing Variability on Network Structures with Spike-Timing-Dependent Plasticity
Source: Front Comput Neurosci. 2018 Jan 23;12:1. doi: 10.3389/fncom.2018.00001 (PMC5787127; doi:10.3389/fncom.2018.00001)
Supplement: Supplementary file 1 [file Presentation1.PDF]

Supplementary Information

October 12, 2017

1 Plastic connections from excitatory neurons to inhibitory neurons

Model

The model we used here is the current-based leaky integrate-and-fire (LIF) model with finite synaptic time constant.

$$\tau_{\mathrm{m}}\frac{dV_i}{dt} = (V_{\mathrm{r}} - V_i) + I_i, \tag{1}$$

$$\frac{dI_i}{dt} = -\frac{I_i}{\tau_{\mathrm{s}}} + \sum_{j=1}^{N_{\mathrm{E}},N_{\mathrm{I}}} w_{ij}s_j(t) + \frac{\mu_{\mathrm{ext},i}}{\tau_{\mathrm{s}}} + \frac{\sigma_{\mathrm{ext},i}}{\tau_{\mathrm{s}}}\sqrt{\tau_{\mathrm{m}}}\xi_i(t), \tag{2}$$

where $\tau_{\mathrm{m}} = 20$ ms is the membrane time constant, $V_{\mathrm{r}} = -60$ mV is the resting potential, $\tau_{\mathrm{s}} = 5$ ms is the synaptic time constant, w_{ij} is the synaptic strength from neuron j to neuron i and $s_j(t)$ is the spike train of neuron j. The parameters $\mu_{\mathrm{ext},i}$ and $\sigma^2_{\mathrm{ext},i}$ are the mean and variance of the external input, respectively, and ξ_i is the white noise satisfying $\langle\xi_i\rangle = 0$ and $\langle\xi_i(t)\xi_j(t^{'})\rangle = \delta_{ij}\delta(t-t^{'})$. Every time when the membrane potential V_i crosses the threshold $V_{\mathrm{th}} = -40$ mV, neuron i would emit a spike and V_i would be reset to V_{r}.

The network consists of N_{E} excitatory neurons and N_{I} inhibitory neurons. We use $N_{\mathrm{E}} = 125$, $N_{\mathrm{I}} = 125$. The connections between these neurons are of the all-to-all type. We keep the inhibitory-to-excitatory (EI) and inhibitory-to-inhibitory (II) synaptic strengths constant during the entire simulation while the excitatory-to-excitatory (EE) and excitatory-to-inhibitory (IE) connections are subject to the STDP learning rule introduced in the main text. At the beginning of simulations, the strengths for the EE and IE are drawn at random from a uniform distribution from 0 mV to $w^{\max}_{\mathrm{EE}} = 2$ mV and $w^{\max}_{\mathrm{IE}} = 4$ mV,

respectively, and the strengths for the EI and II connections are drawn at random from a uniform distribution from $w_{\mathrm{EI}}^{\max} = -8$ mV and $w_{\mathrm{II}}^{\max} = -8$ mV to 0 mV, respectively.

Based on the mean and variance of external inputs, we divide the excitatory neurons into three populations in which the αth ($\alpha = 1, 2, 3$) population receives the external input of the mean $\mu_{\mathrm{ext},\alpha}^{\mathrm{p}}$ and standard deviation $\sigma_{\mathrm{ext},\alpha}^{\mathrm{p}}$. The number of neurons in the αth ($\alpha = 1, 2, 3$) population is denoted as N_α.

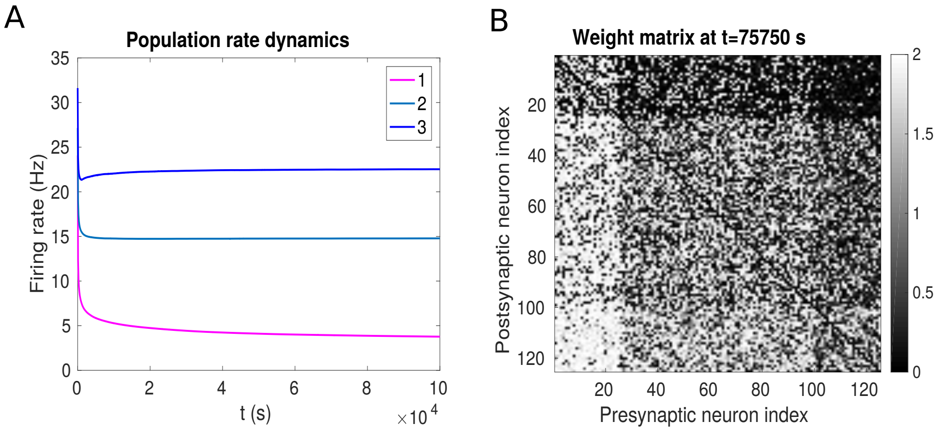

Figure 1: **The effect of firing rate and variability on STDP learning dynamics still holds when STDP is applied on the connections from excitatory neurons to inhibitory neurons.** Different populations receive external inputs with different means as well as different variances. Population 1 (including neurons labeled by 1-25, purple) receives the largest mean ($\mu_{\mathrm{ext},3}^{\mathrm{p}} =$ 32.5 mV) but the smallest variance ($\sigma_{\mathrm{ext},3}^{\mathrm{p}} = 11.2$ mV), Population 2 (including neurons labeled by 25-100, green) receives an intermediate mean ($\mu_{\mathrm{ext},2}^{\mathrm{p}} = 30$ mV) and variance ($\sigma_{\mathrm{ext},2}^{\mathrm{p}} = 22.4$ mV), and Population 3 (including neurons labeled by 101-125, blue) receives the smallest mean ($\mu_{\mathrm{ext},1}^{\mathrm{p}} = 27.5$ mV) but the largest variance ($\sigma_{\mathrm{ext},1}^{\mathrm{p}} = 31.6$ mV) **A**. Firing rate dynamics of the different populations during learning. **B**. Weight matrix after learning. The largest (*i.e.*, brightest) and the smallest (*i.e.*, darkest) value of the grey bar is 2 mV and 0 mV, respectively. We can observe that the connections from Population 1 (the lower firing rate population) to Population 3 (the higher firing rate population) are strengthened while the connections in the opposite direction are weakened.
